# Leveraging Parameter Dependencies in High-Field Asymmetric Waveform Ion-Mobility Spectrometry and Size Exclusion Chromatography for Proteome-wide Cross-Linking Mass Spectrometry

**DOI:** 10.1021/acs.analchem.1c04373

**Published:** 2022-03-11

**Authors:** Ludwig R. Sinn, Sven H. Giese, Marchel Stuiver, Juri Rappsilber

**Affiliations:** † Bioanalytics, Institute of Biotechnology, Technische Universität Berlin, 13355 Berlin, Germany; ‡ Data Analytics and Computational Statistics, Hasso Plattner Institute for Digital Engineering, 14482 Potsdam, Germany; § Digital Engineering Faculty, University of Potsdam, 14469 Potsdam, Germany; ∥ Wellcome Centre for Cell Biology, School of Biological Sciences, University of Edinburgh, Edinburgh EH9 3BF, U.K.

## Abstract

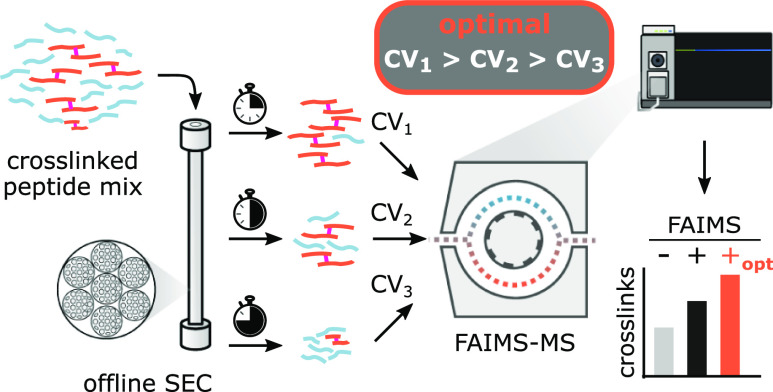

Ion-mobility spectrometry shows great promise to tackle analytically challenging research questions by adding another separation dimension to liquid chromatography–mass spectrometry. The understanding of how analyte properties influence ion mobility has increased through recent studies, but no clear rationale for the design of customized experimental settings has emerged. Here, we leverage machine learning to deepen our understanding of field asymmetric waveform ion-mobility spectrometry for the analysis of cross-linked peptides. Knowing that predominantly *m*/*z* and then the size and charge state of an analyte influence the separation, we found ideal compensation voltages correlating with the size exclusion chromatography fraction number. The effect of this relationship on the analytical depth can be substantial as exploiting it allowed us to almost double unique residue pair detections in a proteome-wide cross-linking experiment. Other applications involving liquid- and gas-phase separation may also benefit from considering such parameter dependencies.

In ion-mobility spectrometry (IMS), ionized analytes are separated in the gas phase based on their individual mobilities within an electric field. Since IMS can operate at near atmospheric pressure with response times in the range of milliseconds, it is widely employed for routine chemical trace analyses such as screening for explosives and other illicit substances in airports or for safety monitoring in the food industry.^1,2^ In addition, differential ion-mobility spectrometry (DMS) increasingly gains attention for its use in life science research.^3−5^ The analyte size, shape, and charge are critical for analyte separation in a commercially available DMS device called FAIMS.^6−8^ However, the relative influences of these and other analyte characteristics for the separation remain a matter of ongoing investigation. Some modified peptides (*e.g.*, SUMOylated or cross-linked) tend to differ exactly in these parameters from the matrix of linear peptides and have thus been targeted through FAIMS^9,10^ and other IMS techniques.^11^

FAIMS is frequently used in conjunction with reversed-phase liquid chromatography–mass spectrometry (LC–MS) to increase analytical sensitivity by improving the overall sample coverage and quantitative accuracy. These benefits are particularly pronounced for investigations of very heterogeneous samples with analyte abundances spanning several orders of magnitude.^12,13^ Here, the LC pre-separates the analyte mixture before subsequent gas-phase separation by FAIMS and mass spectrometric detection. In LC, analyte separation is based on analyte adsorption and/or differential partitioning between a mobile and stationary phase.^14^ As the physicochemical properties of potential analytes are diverse, so are the separation principles in LC to study them—exploiting differences in hydrophobicity, charge state, size, or even subtle steric orientations among others.^15^ It is conceivable that the separation by chromatography and by FAIMS is based on fundamentally different physicochemical analyte properties. However, it may also be that there is an overlap. For example, cross-linked peptides are frequently enriched by size exclusion chromatography (SEC) due to their generally larger size when compared to linear peptides. The hydrodynamic volume that governs separation in SEC is, however, related to the molecular mass and volume, which contribute to FAIMS separation. Importantly, it is therefore possible that for SEC and likely also other chromatographic methods, the optimal settings of FAIMS may vary with the chromatographic fraction under study.

In cross-linking MS, biomolecular structures and interactions are probed *via* locking spatial proximity by newly formed covalent bonds, a process that increases sample complexity.^16,17^ The ultimately obtained cross-linked peptides are separated from the abundantly present linear peptides by chromatographic methods such as strong cation exchange chromatography, SEC, and affinity chromatography relying on tagged cross-linkers.^18−20^ Nonetheless, disentangling cross-linked and linear peptides has so far proven challenging, and substantial improvements are needed. Redefining the coupling of FAIMS to chromatographic methods poses such an opportunity.

In this study, we enhance our understanding of analyte separation in a recent commercial DMS device—FAIMS Pro. We then investigate the interplay between LC-based sample prefractionation and LC-FAIMS-MS for challenging samples that are frequently encountered in cross-linking MS. This leads us to propose revisited settings for FAIMS operation as exemplified with a cross-linked protein complex and an in-cell cross-linked human cell extract when employing SEC-based peptide prefractionation.

## Experimental Section

### Chemicals and Reagents

All reagents were from Sigma (St. Louis, MO; now Merck KGaA, Darmstadt, Germany), unless otherwise stated. Glycerol was from Carl Roth (Karlsruhe, Germany), adenosine 5′-triphosphate (ATP) was from AppliChem GmbH (Darmstadt, Germany), acrylamide (AA) was from VWR International (Dresden, Germany), SPE cartridges were from Empore 3M (Neuss, Germany), bis sulfosuccinimidyl suberate (BS3) and trypsin were from Pierce Biotechnology (Thermo Fisher Scientific, Waltham, MA, USA), and disuccinimidyl sulfoxide cross-linker (DSSO) was from Cayman Chemical (Ann Arbor, MI, USA).

### Cell Culture

293T cells (ACC 635, DSMZ GmbH) were grown in Dulbecco’s modified Eagle medium (DMEM) (1 g/L glucose) with 10% fetal bovine serum. 293T cells expressing C-terminally His6-TEV-biotinylation sequence-His6-(HTBH)-tagged Rpn11 (T6007, Applied Biological Materials Inc.) were grown in DMEM (4.5 g/L glucose) with 10% fetal bovine serum and 2.5 μg/mL puromycin. Cells were cultivated in a humidified, 5% CO_2_ atmosphere at 37 °C.

### Affinity Pull-Down of Human 26S Proteasome

26S proteasomes were isolated according to published protocols.^21^ The lysates were cleared by centrifugation and incubated with streptavidin beads (GE, Cat# 17-5113-01) for 2.5 h at 4 °C. The beads were washed with buffer B (20 mM Hepes-OH pH 7.5, 10% glycerol, 1 mM ATP). Protein was eluted from the beads by TEV protease (Sigma, Cat# T4455-1KU) overnight at 4 °C. The eluate was quantified by the microbicinchoninic acid assay (Thermo Fisher Scientific, Waltham, MA), aliquoted, snap-frozen, and stored at −80 °C.

### Sample Preparation for Protein and Cross-Link Identification

We probed FAIMS with three commonly used cross-linkers, disuccinimidyl suberate (DSS), BS3, and DSSO, and with samples of increasing complexity showcasing common cross-linking MS applications in which FAIMS may be beneficial. (a) Four-protein mix. DSS cross-linker was dissolved in neat dimethyl formamide and added 1:20 (v/v) (to 1 mg/mL DSS) to a solution of human serum albumin, equine myoglobin, chicken ovotransferrin, and cunicular creatine kinase (Roche, Basel, Switzerland), all dissolved in cross-linking buffer (20 mM NaCl, 5 mM MgCl_2_, 20 mM Hepes-OH pH 7.8 at RT) at 1.052 mg/mL each. Proteins were cross-linked for 2 h on ice before adding ammonium bicarbonate (ABC) to 20 mM. Solid urea was added to 8 M. Cysteines were reduced with 5 mM dithiothreitol (DTT) for 30 min at RT followed by alkylation with 15 mM AA for 20 min at RT in the dark. The sample was diluted 1:5 with 50 mM ABC and trypsinized at 25 °C for 16 h [trypsin/substrate of 1:100 (w/w)]. Trifluoroacetic acid (TFA) was added and digest desalted using the Stage-Tip protocol^22^ or using SPE cartridges according to given specifications.

(b) 26S proteasome. A 26S proteasome aliquot was buffer exchanged to buffer C (20 mM Hepes-OH pH 7.8 at 20 °C, 10 mM MgCl_2_, 1 mM ATP, 1 mM DTT) using Amicon Ultra-0.5 mL spin filters with 30 kDa molecular weight cutoff (Merck Millipore, Darmstadt, Germany). Aliquots of the 26S preparation, before and after buffer exchange, were processed as described earlier.^23^ Cross-linking with BS3 was conducted for 2 h on ice at a protein/cross-linker ratio of 1:3.2 (w/w) (to 0.4 mg/mL BS3) until adding ABC to 50 mM (for cross-link titration preexperiment, see Figure S11). The sample was dried and solubilized in 8 M urea and 100 mM ABC and reduced/alkylated. The sample was diluted 1:5 with 100 mM ABC and received trypsin [protease/substrate of 1:50 (w/w)]. After 16 h at RT, another dose of trypsin was added, and incubation was resumed for 2 h at RT. TFA was added, and the digest was desalted using C18-StageTips.^22^

(c) 293T cells. 293T cells were washed twice with 1x phosphate-buffered saline and resuspended in cross-linking buffer (150 mM NaCl, 2 mM MgCl_2_, 0.5 mM DTT, 20 mM Hepes-OH pH 7.8 at 20 °C). DSSO was dissolved in DMF at 50 mM. 5.6 × 10^6^ cells were cross-linked in 0.25 mL of 2 mM DSSO for 45 min at RT until addition of 12.5 μL of 1 M ABC for 15 min at RT (for cross-link titration, see Figure S11). Cells were lysed by 26 μL of 10% (w/v) sodium dodecyl sulfate, 1 M DTT, and 1 M Tris*HCl pH 8.5 at 95 °C for 5 min. Lysates were incubated with 3 units of benzonase at RT for 30 min. The samples were further homogenized using a 26G needle. Cysteines were blocked with AA at 250 mM. Proteins were precipitated by chloroform–methanol as described earlier.^24^ Dried protein pellets were resuspended in 6 M urea/2 M thiourea and 10 mM Hepes-OH at pH 8 and then diluted 1:5 with 100 mM ABC. Trypsin was added at an estimated protease/substrate of 1:50 (w/w) for 18 h at 25 °C until addition of TFA to 0.5% (v/v). Peptides were isolated using C18 SPE cartridges and stored at −80 °C. A non-cross-linked aliquot was processed identically.

### Peptide Fractionation by SEC

Cross-linked peptides were fractionated as described before^19^ using a Superdex 30 Increase 3.2/300 column running with 30% acetonitrile (v/v) and 0.1% (v/v) TFA connected to an ÄKTA Pure system (both Cytiva, Germany). Seven fractions of 50 μL were collected from each run and for each sample. The first two fractions with a low peptide content were pooled to give six fractions of interest. Peptides were dried in a vacuum concentrator (Eppendorf, Hamburg) and stored at −80 °C.

### Analytical Setup—LC–MS

The LC–MS platform consisted of an Ultimate 3000 RSLCnano system (Dionex, Thermo Fisher Scientific, Sunnyvale, USA) connected to a Fusion Lumos Tribrid mass spectrometer (Thermo Fisher Scientific, San Jose, CA) operated under Tune 3.3. Samples were dissolved in 0.1% (v/v) formic acid and 1.6% (v/v) acetonitrile and separated on an EASY-Spray column (50 cm) (Thermo Fisher Scientific) at 300 nl/min flow. 0.1% (v/v) formic acid and 80% (v/v) acetonitrile, and 0.1% (v/v) formic acid were used as mobile phases A and B, respectively. If indicated, the FAIMS Pro IMS device was coupled in between LC and MS, with standard resolution enabled (100 °C for inner and outer electrodes) and no additional gas flow. The emitter tip was placed in a centered position with a distance of *ca.* 1 mm to the entrance plate orifice. For protein identification of the 26S sample preparation, we used an Ultimate 3000 RSLC nano UHPLC coupled to a Q Exactive HF mass spectrometer (Thermo Fisher Scientific, Bremen, Germany) and Tune 2.9.

### Protein Identification by LC–MS

Proteins of 26S proteasome, before and after buffer exchange, were identified to construct sample-specific databases.^25^ Peptides were loaded and separated as described above using the gradient (*t*[min]/B[%]) 0/2, 1/4, 3/6, 75/32.5, 80/37.5, 86/50, 89/90, 96.5/90, 97/2, and 120/2. MS1 spectra were recorded at 120,000 resolution, automated gain control (AGC) target of 3 × 10^6^, maximum injection time (IT) 50 ms, and 350–1600 *m*/*z*. The top ten most intense ions (*z* = 2–6) were isolated within 1.6 Th. Dynamic exclusion was enabled for 30 s. Analyte fragmentation used higher-energy collisional dissociation (HCD) at stepped normalized collision energies (NCEs) of 27, 29, and 31%. MS2 spectra were recorded at 15,000 resolution with an AGC target of 10^5^ and 80 ms maximum IT. The 293T cell proteome was probed using the gradient (*t*[min]/B[%]) 0/2, 2/2, 7/7.5, 87/42.5, 89.5/50, 92/95, 97/95, 98/2, and 121/2. The acquisition regime used 2 s cycles. MS1 spectra were recorded at 120,000 resolution, with 35% source radio frequency (RF), quadrupole isolation between 375 and 1500 *m*/*z*, and normalized AGC target at 250%, and the maximum IT was set to “auto.” MS2 was triggered above an intensity threshold of 5 × 10^3^ and with *z* = 2–6. Dynamic exclusion was set to 30 s. Precursor ions were isolated within 0.4 Th *via* the quadrupole and dissociated by HCD at 30% NCE. Fragment spectra were recorded in the ion trap and operated in the rapid scan mode with an AGC target “standard” and maximum IT “auto.”

### Database Construction for Cross-Link Search

LC–MS data were processed using MaxQuant 1.6.0.16^26^ with default settings, with carbamidomethylation (26S proteasome) or propionylation (293T cells) of cysteines as fixed modification. Quantitation by iBAQ^27^ requiring a minimum of two peptides (unique + razor) and matching between runs were enabled. Uniprot UP000005640 was used as the database [supplemented with TEV protease (P04517) for 26S proteasome]. To facilitate the cross-link search, we constructed a database using not all but only a subset of the proteins that were identified in the sample. We reason that the sensitivity limit for cross-links is much higher than for proteins. We therefore applied a heuristic iBAQ cutoff set around the first inflection point when plotting the sorted iBAQ distribution against all detected proteins. 420 protein groups (26S proteasome) were identified and reduced to 172 entries with at least iBAQ of 10^7^. 5146 protein groups (293T cells) were identified and reduced to 665 entries with at least iBAQ of 7.5 × 10^7^. The four-protein mix database comprised P00563, P68082, P02768, and P02789 without signal peptides.

### Cross-Link Detection by LC–MS ± FAIMS Pro

The cross-linked four-protein mix was separated using the gradient (*t*[min]/B[%]) 2/2, 7/7.5, 87/45, 89.5/52.5, 92/95, 97/95, 98/2, and 121/2. For the 26S proteasome and 293T cells, LC gradients were adjusted for each SEC fraction. The gradients were 0/2, 10/9–22, 90/40–55, 92.5/55–60, 95/95, 100/95, 101/2, and 119/2. MS settings for the four-protein mix were 2100 V emitter voltage, data-dependent acquisition with 2 s cycle time, MS1 spectra at 120,000 resolution, quadrupole isolation from 400 to 1450 *m*/*z*, source RF 35%, AGC target 250%, and maximum IT set to “auto”; for MS2 spectra, precursor charge filter *z* = 3–7^+^ (4–7^+^ prioritized) and intensity threshold is 2.5 × 10^4^. Precursors were isolated using the quadrupole within 1.4 Th, AGC target “standard,” and the maximum IT “dynamic.” Precursors were subjected to HCD with data-dependent decision tree logic.^28^ MS2 spectra were recorded at a resolution of 50,000. Measurements with the four-protein mix were duplicated using FAIMS with external stepping compensation voltages (CVs) of −20, −25, −30, −35, −40, −45, −50, −55, −60, −65, −70, −75, −80, −85, −90, −100, and −110 V.

For the 26S proteasome and 293T cells, the same settings were used except 2000 V emitter voltage, 2.5 s cycle time, quadrupole isolation from 400 to 1500 *m*/*z* (26S sample), maximum IT for MS1 50 ms; for MS2 resolution 60,000, quadrupole precursor isolation within 1.6 Th (26S sample), dynamic exclusion ±10 ppm for 60 s. For 293T cells, AGC target 200% and maximum IT 118 ms were used. MS2 range was 150–2000 *m*/*z*. Precursors were fragmented with stepped NCEs of 18, 24, and 30%.^23^ Each measurement used an internal stepping CV pair as follows: −30/–60, −35/–65, −40/–70, −45/–75, −50/–80, and −55/–85 V. A 30 V offset was used to pair two CVs. We paired CVs such that we combined one CV leading to more precursors with one that led to fewer precursors to reduce the effect of time restriction on precursor selection for comparing individual CV values. The dwell time for each CV was dependent on the duty cycle of the mass analyzer and ranged up to 2 s depending on the observed precursor ions. Each SEC fraction was acquired in six LC-FAIMS-MS runs using one out of six CV pairs. Also, SEC fractions were measured in triplicate without FAIMS.

### Cross-Linking Data Processing and Analysis

The raw LC–MS data were split by CV using Freestyle 1.6 and converted to mgf-file format using msConvert^29^ (version 3.0) including denoising (DSS/BS3 data only, top 20 peaks in 100 *m*/*z* bins).^30^ Peak files were searched with xiSEARCH 1.7.6.1^31^ with MS1/MS2 matching tolerances 2 and 5 ppm, up to 2 missing isotope peaks,^30^ up to 2 missed cleavages, with propionylation on cysteine as fixed and oxidation on methionine as variable modifications. Losses of methanesulfonic acid, water, and ammonia were considered. Cross-linker modifications were defined as variable.^23^ For 293T cells, variable cross-linker modifications were only considered for linear peptides. Cross-linker specificities were defined as reacting with Lys, Tyr, Ser, Thr, and the peptide N-terminus. Methylation of Glu was set as variable modification for linear peptides. Loss masses were enabled to account for the DSSO linker cleavage.^23^ A “noncovalent cross-linker” with zero mass was defined to flag the spectra putatively arising from gas phase-associated peptides, which were removed from the list of spectra prior to false discovery rate (FDR) estimation.^32^ Search results were filtered prior to FDR estimation to cross-link spectrum matches (CSMs) with a minimum of three matched fragments per peptide (two for 26S proteasome) and a delta score ≥0.1. Each dataset, that is, with and without using FAIMS, was individually filtered to an FDR of 1% on CSM level using xiFDR (version 2.1.3).^33^ The minimal peptide length was set to 5, and CSM redundancy was allowed. FDR-filtered results were processed using python 3.7 with pandas 0.24.2 and numpy 1.16.2.^34^ Plots were created with python using the seaborn 0.9.0 package.

### Machine Learning on FAIMS-Assisted Separation of Cross-Linked Peptide Data

For the supervised machine learning prediction of CV values for cross-linked peptides, we used data from our DSS-cross-linked four-protein mix and an eight-protein mix from data set PXD019926,^10,35^ both filtered to 1% CSM–FDR. Despite the discrete nature of the CV distribution, we modeled the prediction problem as a regression task. The combined CSMs (*n* =10, 119) were further reduced to only include the highest scoring peptide pair from combinations of the alpha-peptide, beta-peptide, and charge state. This allows a conservative training set generation, without sequence information leakage arising from different linkage sites within the same peptide. The resulting 4431 target–target CSMs were divided into a training set (80%, 3544 CSMs) and a validation set (20%, 887 CSMs). The training set was further used in a three-fold cross-validation grid search including regularization to optimize the hyper-parameters of XGBoost (version 1.1.1) while minimizing the negative mean-squared error. The grid search included 1152 parameter combinations (Table S1) and 32 features (Table S2).^36,37^ For the interpretation of the learned tree model, we used the tree explainer from the SHAP package (version 0.36.0).^38^ SHAP values were computed for the validation data. The code is available on GitHub (https://github.com/Rappsilber-Laboratory/xiFAIMS).

### Data and Code Availability

The mass spectrometric raw data, peak lists, mzid result tables, and used FASTA databases have been deposited with the ProteomeXchange Consortium (http://proteomecentral.proteomexchange.org) *via* the jPOST partner repository,^39^ JPST000990/PXD022341 (protein identification), and JPST000989/PXD022360 (cross-linking MS).

## Results and Discussion

### FAIMS Pro Separation is Predominantly Influenced by the Mass-to-charge Ratio, Size, and Charge State of Analytes

**Figure 1:**
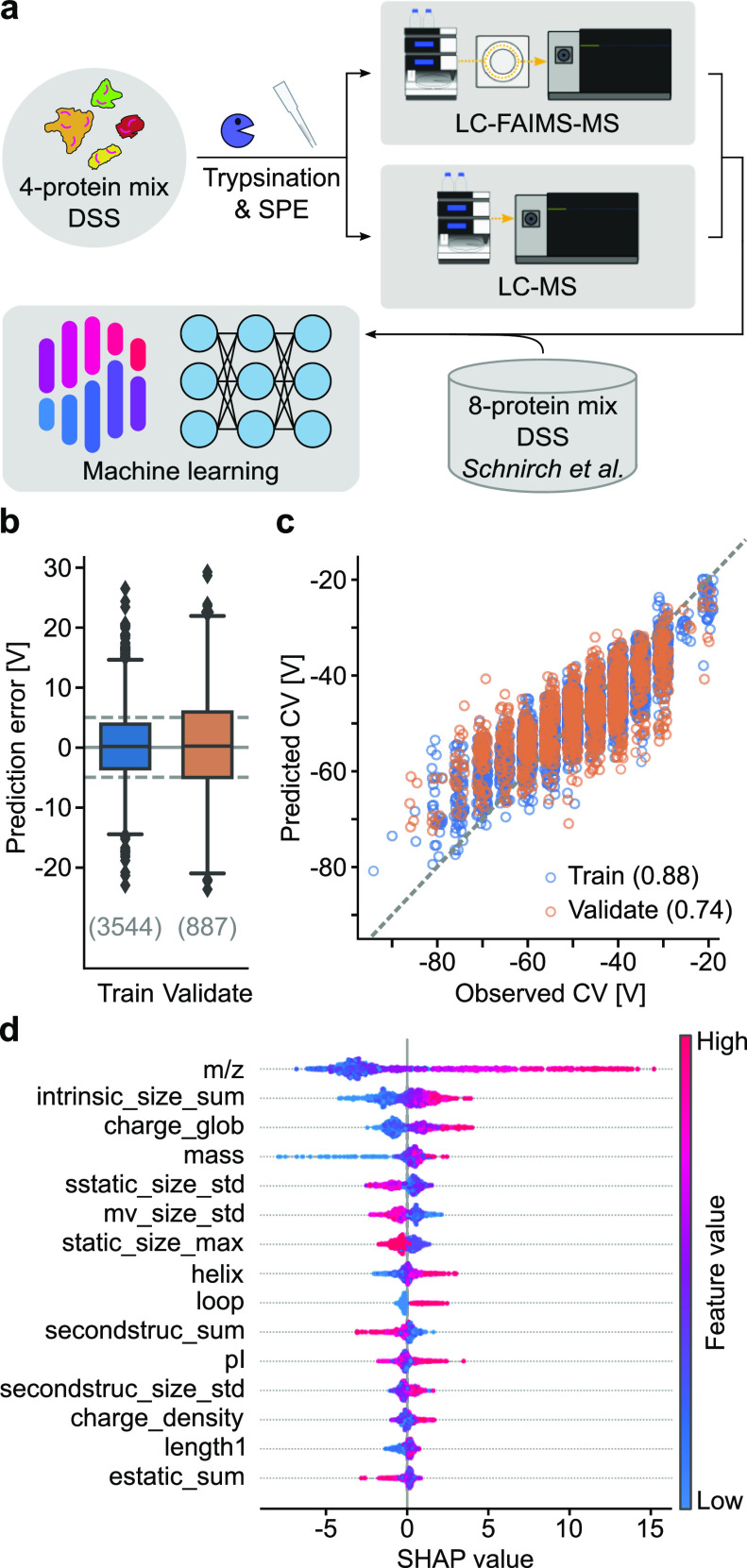
Machine learning-assisted characterization of cross-linked peptide separation with FAIMS Pro. (a) Experimental workflow. Data from a DSS-cross-linked four-protein mix analyzed by LC-FAIMS-MS or LC–MS were merged with a dataset from Schnirch *et al.*(10) and subjected to explainable machine learning. (b) Machine learning prediction performance on training and validation data subsets (see Methods for details). Dashed line corresponds to ±5 V error margin. Numbers in brackets indicate the number of data points for each set. (c) Prediction accuracy on training and validation data subset-based CV prediction error. Numbers in brackets represent the Pearson correlation coefficient for each data subset. (d) Global feature importance for differential ion-mobility prediction from the SHAP value analysis. The 15 most important features are shown (declining impact from top to bottom). The feature-specific impact on the prediction is shown on the *x*-axis. The color gradient illustrates the relative value distribution of a corresponding feature for each CSM (represented by a dot).

In analogy to previous studies,^28,40,41^ we tested our analytical setup with a digest of four proteins which were individually cross-linked with the noncleavable cross-linker DSS (Figure 1a) prior to analysis using a FAIMS Pro device coupled between LC and an orbitrap mass spectrometer. In total, we detected 9100 CSMs at 1% CSM–FDR (2731 unique) with FAIMS using single compensation voltages (CVs) incrementing by 5 V across the range −100 to −20 V in two series of 17 runs. Overall, the observed distribution matched what others had observed before (Figure S1).^10^ Notably, using FAIMS at a single CV did not outperform the analysis without FAIMS.

The applied CV values generally correlated with the mass-to-charge ratio and the size of a given cross-linked peptide, while the charge in the observed range of *z* = 3–7^+^ played a minor role. This agrees with previous observations made for linear and cross-linked peptides^6,10,42^ (Figure S1). However, to learn about peptide features governing separations with FAIMS Pro more systematically and to possibly predict them, we employed an explainable machine learning approach.^43^ We manually defined a broad set of physicochemical peptide features (Table S1) to be used by XGBoost on a combined dataset comprising our data on a DSS-cross-linked protein mix and that of a similar study on eight DSS-cross-linked proteins.^10^ XGBoost was optimized by minimizing the negative mean-squared error during the hyperparameter grid search in a 3-fold cross-validation approach (Figure S2). In addition, we used the absolute prediction error as the evaluation metric (Figure 1b). About 47% of the data was predicted correctly within a margin of ±5 V and 78% within ±10 V. Overall, the prediction performance turned out moderate-to-fairly strong with Pearson’s correlation coefficients of 0.88 for the training data and 0.74 for the validation data (Figure 1c). This discrepancy in prediction accuracy between training and validation data occurred despite including regularization means during the grid search. A confounding factor might result from unconsidered features during the machine learning (Figure S3 and Supporting Information discussion). Indeed, a subset of peptides, larger peptides with high charge states, could be predicted better than others. Nonetheless, our model learned features that agree with previous findings of analyte behavior.^7,44^

*Via* SHAP value analysis on the features underlying the learned model, again we found the *m*/*z* of a peptide to be the most impactful feature for predicting its separation behavior (CV value) with FAIMS Pro, with the analyte size following as the second and the calculated peptide charge state in solution being the third (Figure 1d). Since mass and charge are essential parameters needed to describe acceleration in an electrical field, *m*/*z* likely turned out to be the strongest feature for the prediction because it combines these two parameters (following as third and fourth). Interaction with gas molecules also plays a role, which was reflected by the second most important parameter returned, the size.

### Optimal FAIMS CV Settings Depend on the SEC Fraction under Study

In the field of cross-linking MS, SEC is frequently used to enrich cross-linked peptides over linear peptides.^19,31,45^ Since cross-linked peptides tend to be larger than linear peptides, they generally elute earlier during SEC. Size being a factor also influencing FAIMS separation suggests that optimal FAIMS CV settings and SEC fraction number might be dependent parameters. We tested this by separating a cross-linked peptide mixture *via* SEC and probed each fraction using LC-FAIMS-MS with different CV combinations.

**Figure 2:**
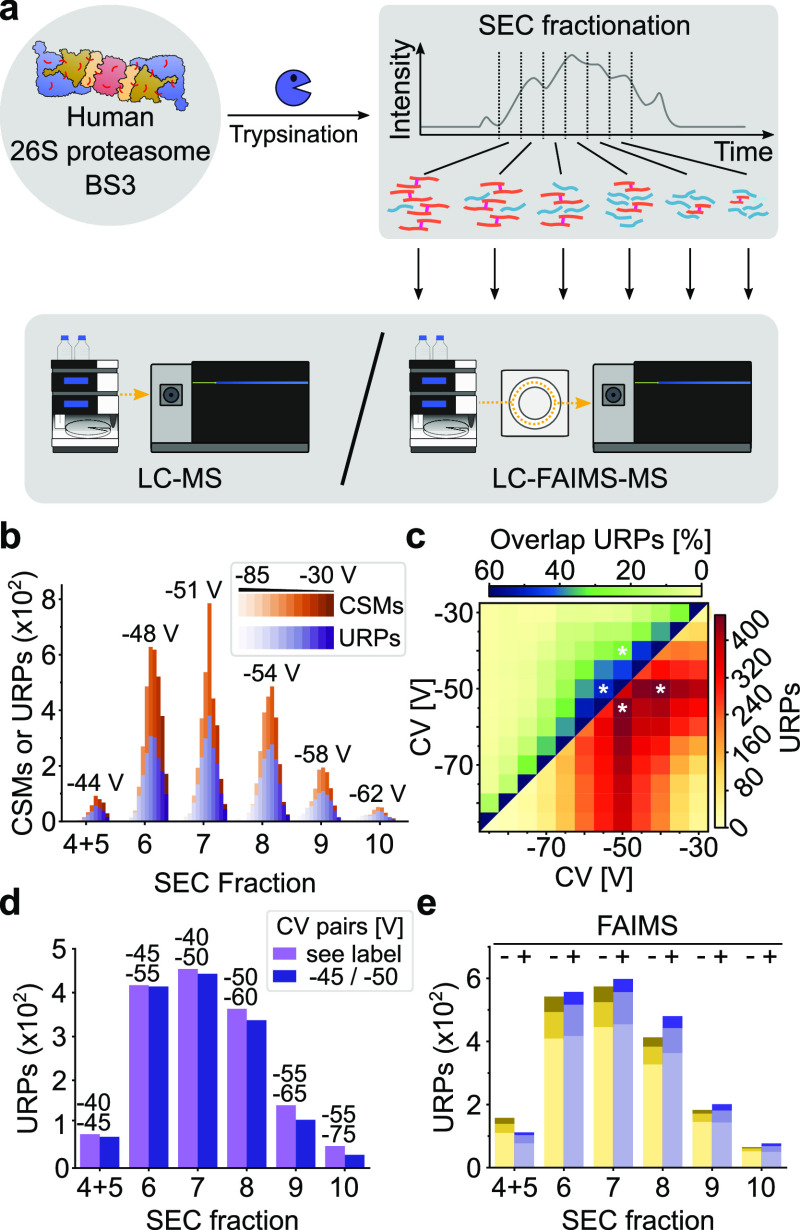
FAIMS-assisted LC–MS for the SEC-fractionated 26S proteasome*BS3 sample. (a) Experimental workflow to probe FAIMS separation parameter dependency from SEC fractionation. The 26S proteasome was cross-linked using BS3, tryptically digested, and fractionated by SEC, leading to six fractions of interest. The analysis by LC–MS was conducted with or without the FAIMS Pro ion-mobility device coupled in between LC and MS. (b) Histogram of detected CSMs and URPs for each CV value, split by the SEC fraction. Values indicated on the top represent the average CV for a given SEC fraction. A trend to more negative CVs was observed with later SEC fractions. (c) Heatmaps on combining two CVs for SEC fraction 7 (for fraction-wise and global heatmaps, see Figures S5 and S7). In the top left, the percentaged overlaps in URPs; in the bottom right, the sum of URPs from a 2-CV combination are given. Asterisks mark the best 2-CV combinations for this fraction. (d) Histogram of detected URPs comparing fraction-wise (“see label”) and global (“–45/–50”) optimal 2-CV-combinations with the CV value pairs indicated. (e) Histogram on achieved gains in identifications of URPs upon repeated measurements (one to three, stacked from the bottom to top) when using FAIMS (blue) compared to not using it (yellow). For FAIMS-assisted measurements, 2-CV combinations maximizing identifications were merged *in silico*.

First, we cross-linked affinity-purified human 26S proteasome^21^ with the noncleavable cross-linker BS3. Tryptic peptides were separated by SEC yielding six fractions of interest, following a standard procedure used for in-depth cross-linking MS analysis of cross-linked protein complexes^46^ (Figure 2a). We acquired FAIMS-assisted LC–MS data with internal stepping between two CVs over the separations lasting for 2 h each, as was done before for a similar sample type.^10^ We sampled CVs ranging from −30 to −85 V in 5 V increments, as this range covered most peptides in the analysis of our cross-linked four-protein mix (see above). To mitigate potential biases arising from cycle time restrictions from the data-dependent acquisition regime, we aimed to create balanced CV pairs. We paired one peptide-rich CV with one peptide-poor CV value to give six pairs with a constant difference of 30 V between the individual CVs. Every fraction was additionally acquired in triplicate without using FAIMS. In total, we detected 10,477 CSMs (2733 unique) with FAIMS and 10,357 CSMs (2473 unique) without using FAIMS at 1% CSM–FDR. Most CSMs were observed at a CV of −50 V, in accordance with published data.^10^ In the first analyzed fraction (fraction 5) the number of CSMs and unique residue pairs (URPs) peaked around an average of −44 V. This peak CV value changed successively for the subsequent SEC fractions to reach −62 V for the last analyzed fraction (fraction 10) (Figure 2b). These data revealed a dependency between the optimal CV value and a given SEC fraction. However, the number of CSMs and URPs identified without FAIMS surpassed the numbers when using FAIMS for all individual CV values, despite FAIMS’ assumed capability to result in more unique CSMs (Figure S4).

Note that multiple CV values can be combined in one LC-FAIMS-MS analysis. We therefore investigated if the results for FAIMS improved when combining two CVs for a given SEC fraction *in silico*, as exemplified for fraction 7 (Figure 2c). For fraction 7, an exhaustive pairwise combination of our CV values revealed that the CV value pair of −50/–55 V yielded most URPs. This was the case despite 50% overlap in URPs between these two values. Optimal CV value pairs existed also for the other fractions (Figure S5). In agreement with the observation of the CV value dependence on the fraction number, also the pair values rose from −40/–45 V (or −45/–50 V) for fraction 5 to −55/–80 V (or −60/–80 V) for fraction 10. The large spacing of CV values for fraction 10 being the exemption, best CV pairs were spaced by 5–10 V, despite the resulting large overlap in CSMs (Figure S5). The narrow spacing results from the confined CV range of −40 to −60 V in which most CSMs are observed (Figure S1). This is also underpinned by a peak in ion transmission in this CV value range (Figure S6).

Given fraction-specific CV pairs maximizing URP detection, we wondered how a single and more general CV pair would compare. Using the same approach as before but now for the entire dataset, we obtained −45/–50 V as the optimal CV pair for the BS3-cross-linked proteasome sample (Figure S7). The gain of using fraction-specific CV pairs increased as the difference between CV values increased between the global and fraction-specific settings, peaking for fraction 10 at a gain of +67% from using the fraction-specific CV value pair (Figure 2d).

Next, we investigated how many more URPs could be observed if one had up to three injections on LC-(FAIMS)-MS per fraction. We extracted the three best CV pairs for each fraction and compared their URPs to technical triplicate acquisitions without FAIMS. We found improvements for all fractions other than fraction 4 + 5 (Figure 2e) of 7 ± 17.3% (median ± std). The benefit of using FAIMS, however, was lower than what others had described (about +60%).^10^ This moderate gain by using FAIMS might result from a moderate dynamic range of cross-linked peptides in our sample after enrichment through SEC prefractionation. We therefore investigated the gains of FAIMS in the context of a more complex sample next.

### SEC Prefractionation and FAIMS Pro Excel for a Complex Sample

**Figure 3:**
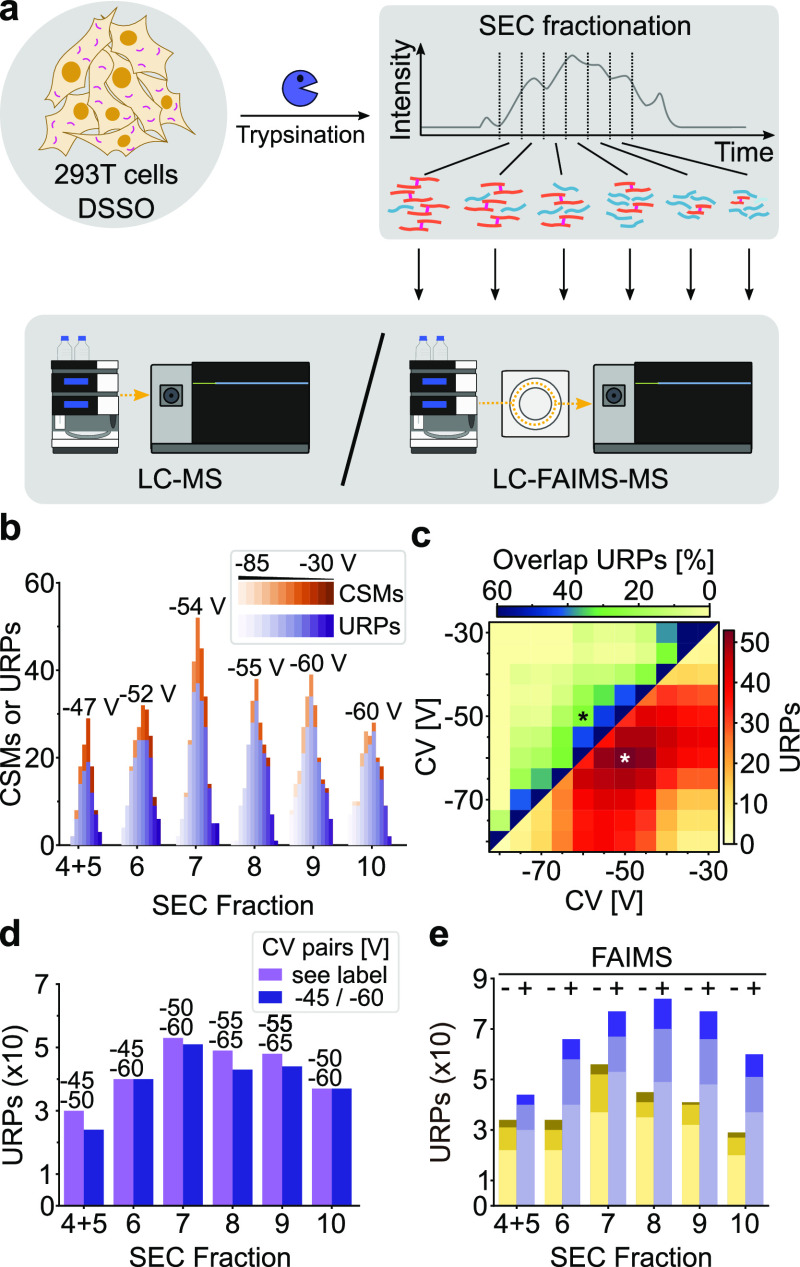
FAIMS-assisted LC–MS for the SEC-fractionated 293T*DSSO sample. (a) Experimental workflow to probe FAIMS separation parameter dependency from SEC fractionation. 293T cells were cross-linked *in situ* using DSSO, tryptically digested, and fractionated by SEC, leading to six fractions of interest. The analysis by LC–MS was conducted with or without the FAIMS Pro ion-mobility device coupled in between LC and MS. (b) Histogram of detected CSMs and URPs for each CV value, split by the SEC fraction. Values indicated on the top represent the average CV for a given SEC fraction. A trend to more negative CVs was observed with later SEC fractions. (c) Heatmaps on combining two CVs for SEC fraction 7 (for fraction-wise and global heatmaps, see Figures S9 and S10). In the top left, the percentaged overlaps in URPs; in the bottom right, the sum of URPs from a 2-CV combination are given. The asterisks mark the best 2-CV combination for this fraction. (d) Histogram of detected URPs comparing fraction-wise (“see label”) and global (“–45/–60”) optimal 2-CV combinations with CV value pairs indicated. € Histogram on achieved gains in identifications of URPs upon repeated measurements (one to three, stacked from the bottom to top) when using FAIMS (blue) compared to not using it (yellow). For FAIMS-assisted measurements, 2-CV combinations maximizing the identifications were merged *in silico*.

To test our analytical setup with a sample at the level of cellular complexity, we cross-linked intact human embryonic kidney cells 293T with the cleavable cross-linker DSSO. Tryptic peptides were fractionated *via* SEC, and the resulting fractions were acquired on LC-(FAIMS)-MS alike to the 26S proteasome sample (Figure 3a). We detected 1135 CSMs (465 unique) with FAIMS and 581 CSMs (255 unique) without using FAIMS after filtering to an FDR of 1% at the CSM level. We found −55 V as the best single CV. This differs from the best CV observed above for BS3 by −5 V (Figures S4 and S8). However, these values agree with a previous study using DSSO and DSS cross-linkers.^10^

As with the proteasome sample, peak CVs shifted to more negative values along the course of fractionation, ranging from −47 to −60 V, and thus confirming our findings on a dependency between the optimal CV value and a given SEC fraction (Figure 3b). In contrast to the proteasome sample, however, we observed more CSMs and URPs when using FAIMS at a CV of −55 V compared to measurements without FAIMS (Figure S8). For each fraction, some pair of CVs maximized the number of URPs, for example, CV −50/–60 V for fraction 7 (Figure 3c).

As was the case for the proteasome sample, increasingly negative CV value pairs yielded best results with increasing fraction numbers, albeit the optimal CV value pairs for individual fractions did not vary as much (Figure S9). This trend was true for fractions 5–9 (−45/–50 V and −55/–65 V), while fraction 10 represented an exemption with −50/–60 V. Again, CV value pairs spaced by 10 V maximized the number of URPs. The overlaps of detected URPs from exhaustive CV combinations for each SEC fraction were lower than those from the proteasome sample, as can be expected from a sample with greater underlying complexity.

Since the best-found CV pairs did not vary much for this sample, we reasoned that a single CV pair might be suitable to arrive at a reasonable number of URPs from all fractions (Figure S10). Yet, comparing the global optimum CV pair of −45/–60 V with fraction-specific CV pairs revealed gains of 6.5 ± 9.7% (median ± std) with up to +25% for individual fractions when using the fraction-specific CV pairs (Figure 3d).

Finally, we examined how much more URPs one can expect from up to three injections on LC-(FAIMS)-MS for each fraction following the procedure introduced above. Strikingly, we achieved URP improvements for all fractions with a median of 85 ± 31.8% (std) for this sample (Figure 3e). LC-FAIMS-MS thus enabled us to almost double the number of URPs from a SEC-prefractionated sample of cellular complexity, thereby illustrating the added value of FAIMS. Note that even a single LC-FAIMS-MS acquisition employing the best fraction-specific CV value pair outperformed triplicate injections without FAIMS for most fractions tested, with a median of +13%. Multiple measurements that use different FAIMS settings could consequently add many more unique CSMs than replica.

## Conclusions

FAIMS—and also other forms of IMS—probe peptide properties also probed by hyphenated separation technologies such as SEC, with smaller peptides eluting from SEC later and passing FAIMS at more negative CVs. This leads to an interdependency of measurement parameter choices. Leveraging this observation substantially improved the analytical outcome for whole cell cross-linking, an analytically challenging sample with an extreme underlying dynamic range. Of note, for this sample even a single injection with FAIMS typically outperformed triplicate measurements without FAIMS. When comparing triplicate analyses, FAIMS almost doubled the number of observed links. Importantly, this may extend to other chromatographic methods. For example, retention on reversed-phase chromatography is influenced by parameters such as the analyte volume or surface^47^ which also influence the behavior of analytes within FAIMS. In consequence, optimal CV values might change during the prefractionation of peptides by high pH reversed-phase chromatography^48^ or even during the separation of peptides by reversed-phase LC–MS. When linked *via* analyte properties, FAIMS and chromatography form a joint workflow where the parameter choice on one end influences the parameter choice on the other end.

## Supporting Information Available

Additional experimental details and an extended characterization of FAIMS separation of cross-linked peptides (PDF)
