# Supplementary material for: Leveraging Parameter Dependencies in High-Field Asymmetric Waveform Ion-Mobility Spectrometry and Size Exclusion Chromatography for Proteome-wide Cross-Linking Mass Spectrometry
Source: Anal Chem. 2022 Mar 11;94(11):4627–34. doi: 10.1021/acs.analchem.1c04373 (PMC8943524; doi:10.1021/acs.analchem.1c04373)
Supplement: Supplementary file 1 — ac1c04373_si_001.pdf [file ac1c04373_si_001.pdf]

# Supporting Information: Leveraging Parameter Dependencies in High-Field Asymmetric Waveform Ion-Mobility Spectrometry and Size-exclusion Chromatography for Proteome-wide Crosslinking Mass Spectrometry

Ludwig R. Sinn[1], Sven H. Giese[1,2,3], Marchel Stuiver[1] and Juri Rappsilber[*,1,4]

1 Bioanalytics, Institute of Biotechnology, Technische Universität Berlin, 13355 Berlin, Germany

2 Data Analytics and Computational Statistics, Hasso Plattner Institute for Digital Engineering, 14482 Potsdam, Germany

3 Digital Engineering Faculty, University of Potsdam, 14469 Potsdam, Germany

4 Wellcome Centre for Cell Biology, School of Biological Sciences, University of Edinburgh, Edinburgh EH9 3BF, United Kingdom

*Corresponding author: juri.rappsilber@tu-berlin.de

**CONTENTS**

**Figure S-1: Overall characterization of FAIMS for the separation of crosslinked peptides**

**Figure S-2: Assessment of prediction quality from machine learning by negative mean squared error**

**Figure S-3: Crosslinked peptides with m/z < 900 show a more complex separation behaviour with FAIMS**

**Supplemental discussion to Figure S-3**

**Figure S-4: Unique CSMs and links from +/-FAIMS for 26S*BS3 sample in dependence of CV and in total**

**Figure S-5: Optimal CV pairs for individual SEC fractions of 26S*BS3 sample**

**Figure S-6: Distribution of MS1-features in dependence on CV during FAIMS separation with 26S*BS3 sample**

**Figure S-7: Optimal CV pair for unique CSM and residue pair detection for all SEC fractions of 26S*BS3 sample**

**Figure S-8: Unique CSMs and links from +/-FAIMS for 293T*DSSO sample in dependence of CV and in total**

**Figure S-9: Optimal CV pairs for individual SEC fractions of 293T*DSSO sample**

**Figure S-10: Optimal CV pair for unique CSM and residue pair detection for all SEC fractions of 293T*DSSO sample**

**Figure S-11: Crosslinker titrations with 26S*BS3 and 293T*DSSO**

**Table S-1: XGBoost regression parameters used in a 3-fold cross-validation grid search**

**Table S-2: Feature set for supervised machine learning**

**References**

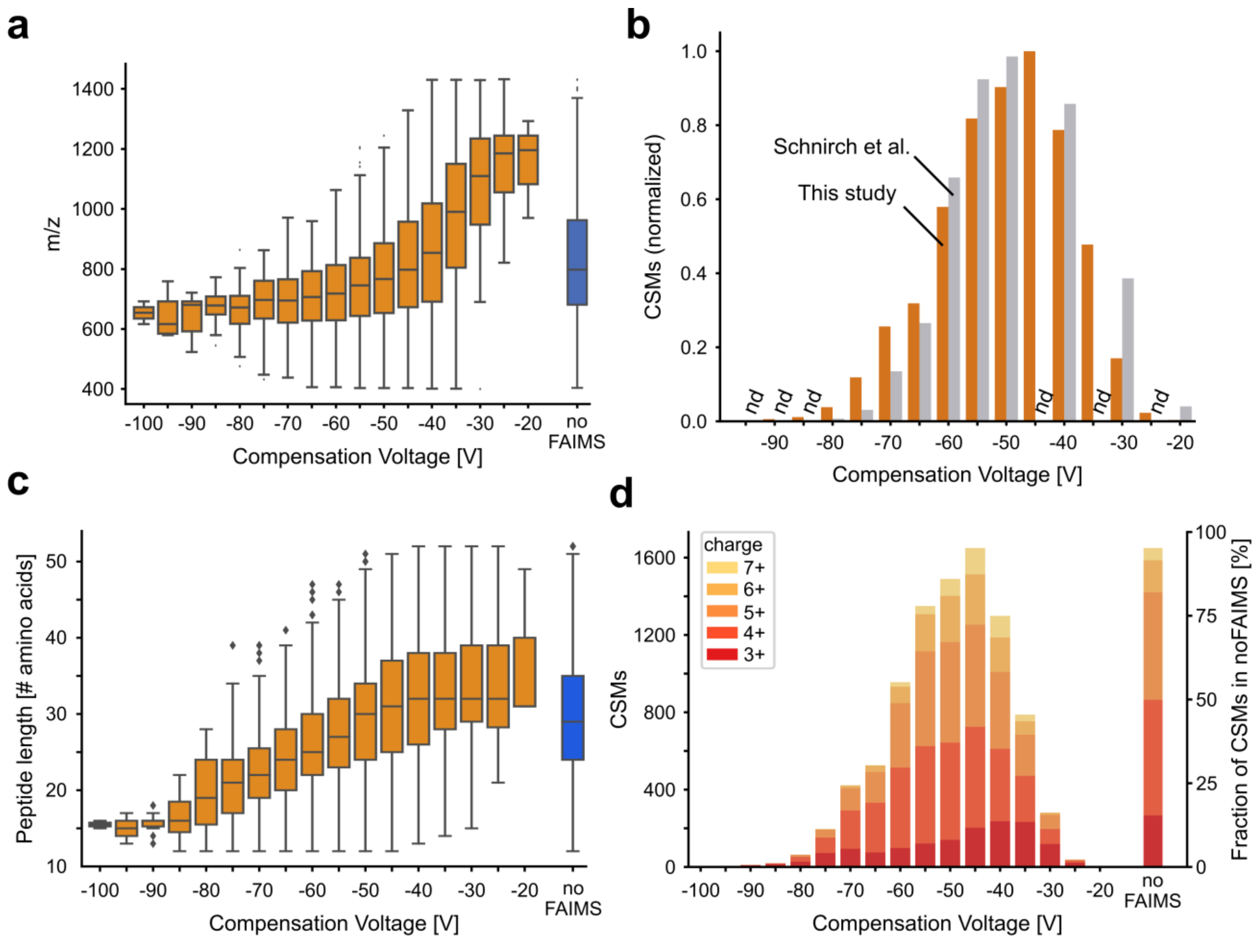


**Figure S-1: Overall characterization of FAIMS for the separation of crosslinked peptides**
FAIMS separation of DSS-crosslinked peptides from the 4-protein mix is dependent on m/z (a), overall peptide size (c) and charge state (d). A comparison to not using FAIMS is given at the right of each plot. Panel (b) displays the normalized counts of CSMs at a given FAIMS compensation voltage comparing results from DSS-crosslinked protein standards between Schnirch et al.[1] and this study. Compensation voltages at which no data was determined by Schnirch et al. [1] are indicated as "nd". Abbreviation: CSMs = crosslinked spectrum matches.

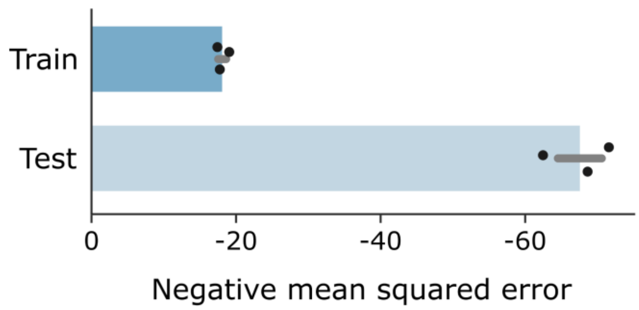


**Figure S-2: Assessment of prediction quality from machine learning by negative mean squared error**
Machine learning prediction performance on training and training data subsets based on a negative mean square error loss function. A higher value for the Testing compared to the Training data subset indicates overfitting by the trained XGBoost classifier.

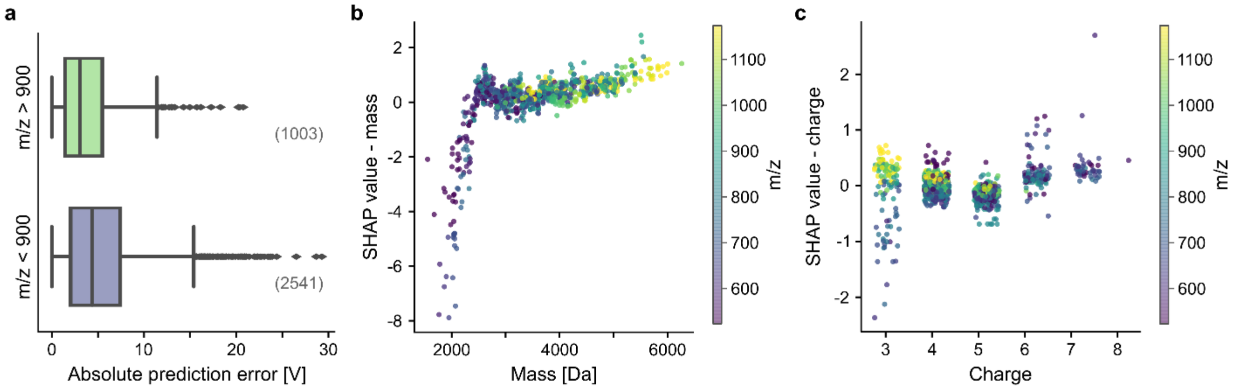


**Figure S-3: Crosslinked peptides with m/z < 900 show a more complex separation behaviour with FAIMS**
a) Absolute prediction errors for analytes above and below 900 m/z. The number of data points for each subset is given in brackets. Analytes below 900 m/z show a larger absolute prediction error. b, c) Feature interaction between (b) analyte mass and m/z, and (c) observed charge state and m/z within the prediction. For analytes below 2.5 kDa the mass feature gains predictive value towards more negative CV values (b). Crosslinked peptides with z=3+ and low m/z influence the prediction towards more negative CV values (c).

## Supplemental discussion to Figure S-3

A closer inspection of the prediction quality revealed that its accuracy was related to m/z (Figure S-3). For crosslinked peptides with m/z < 900 the absolute prediction error was larger than for those with m/z > 900 (4.38 compared to 3.05 V abs. deviation) despite having more training data for analytes with m/z below 900 than above (2,541 compared to 1,003 CSMs). Also, we noticed that the subset of crosslinked peptides with m/z < 900 deviated from those of other peptides in SHAP values when considering mass (< 2.5 kDa) and charge state (predominantly z=3+) (Figure S-3). Our machine learning model thus learned some specific aspects of the m/z < 900 subset. However, the prediction accuracy for this subset did not benefit from that knowledge. Consequently, we likely miss features describing some properties of crosslinked peptides with m/z < 900. Predicted secondary structure elements of the individual peptides only had a minor influence on predicting FAIMS separation of crosslinked peptides (Figure 1d). However, secondary structure could also form across the two peptides of a crosslinked peptide. We suspect currently unconsidered interactions between the two crosslinked peptides to be a relevant missing feature. This has also been proposed to play a role in the ion mobility of SUMOylated peptides[2], which constitute another type of branched peptide. At higher charge states and larger analyte size, these interactions may decrease in relative effect. Because of branching, recent findings on linear peptides may be insufficient to explain the observed behaviour[3].

The role of branching of crosslinked peptides during FAIMS separation might be captured for machine learning by molecular dynamics simulations. These may have to consider the influence of analyte desolvation and binding of gas molecules on the conformation space of a crosslinked peptide. Since these processes are affected by the electrical field and vacuum gradients (basis of DIMS separation), the computation is not straightforward and warrants an independent investigation. In any case, crosslinked peptides with m/z > 900 can be predicted with higher accuracy. Only considering these crosslinked peptides in the validation data subset (258 of 887 CSMs), about 56% of the data was predicted correctly within a margin of +/-5 V (compared to 47% for all m/z values), and 90% within +/-10 V (compared to 78% for all m/z values).

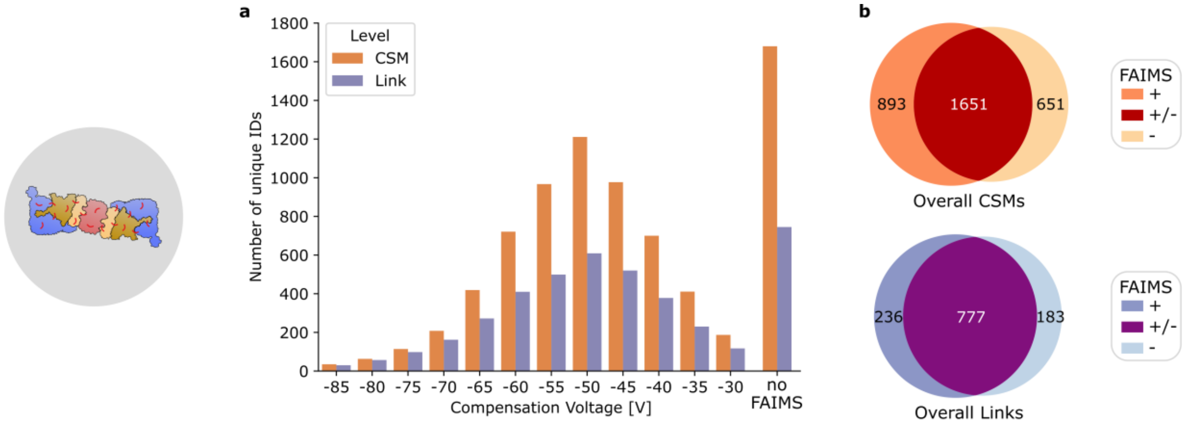


**Figure S-4: Unique CSMs and links from +/-FAIMS for 26S*BS3 sample in dependence of CV and in total**
(a) FAIMS separation of BS3-crosslinked peptides from the 26S proteasome displays a unimodal relationship between detectable CSMs and compensation voltage with an optimum at -50 V. Each bar represents the outcome from a single LC-FAIMS-MS analysis considering one CV or the median from triplicate LC-MS analyses of two hour analysis time both, respectively. Using FAIMS led to less detected CSMs and crosslinked residue pairs than not using it. (b) Venn diagrams on overall uniquely detected CSMs and links with or without using FAIMS. Abbreviation: CSMs = crosslinked spectrum matches.

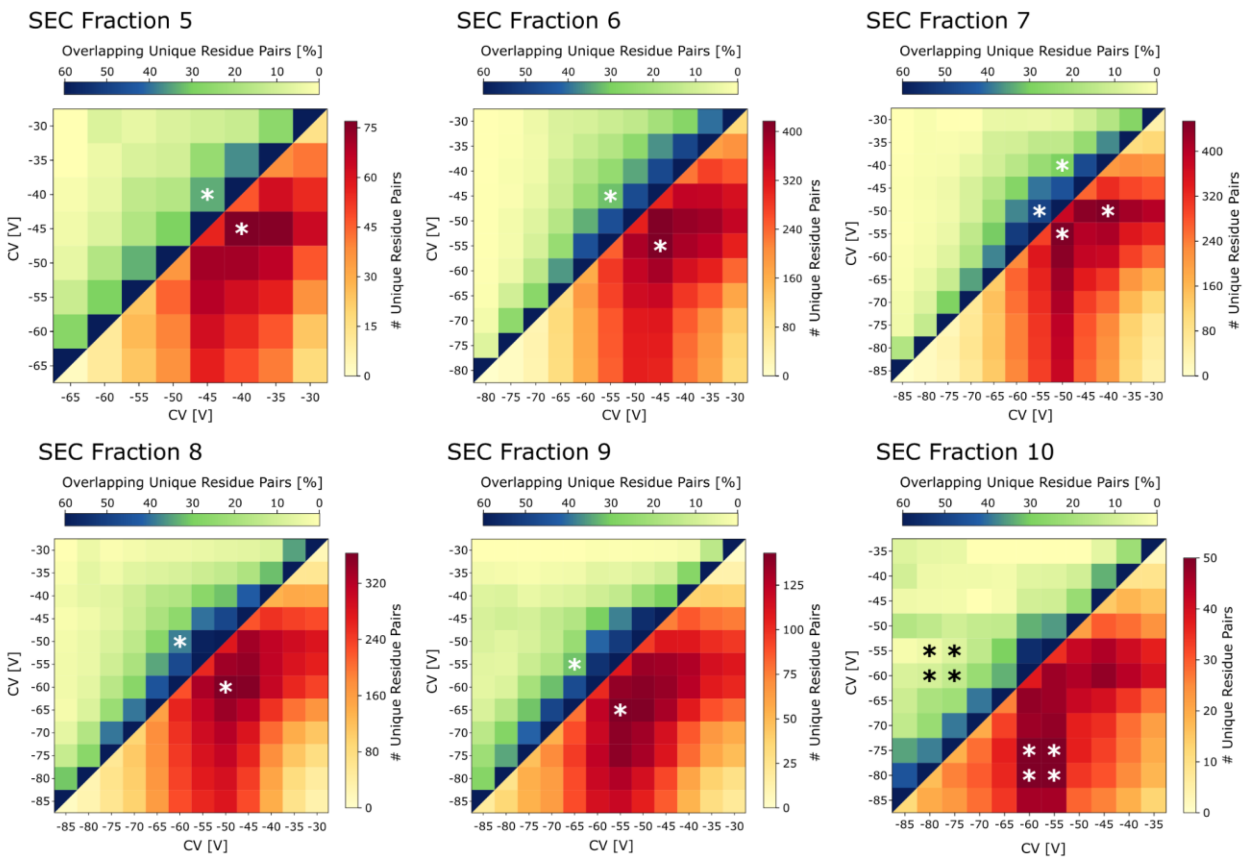


**Figure S-5: Optimal CV pairs for individual SEC fractions of 26S*BS3 sample**

The heat maps show the result from combining two compensation voltages in-silico on the detected number of unique residue pairs and their corresponding overlaps for the 26S proteasome crosslinking experiment, split by each SEC fraction. Asterisks (white or black) mark the best combination(s) of two CV values on both sections of the plot.

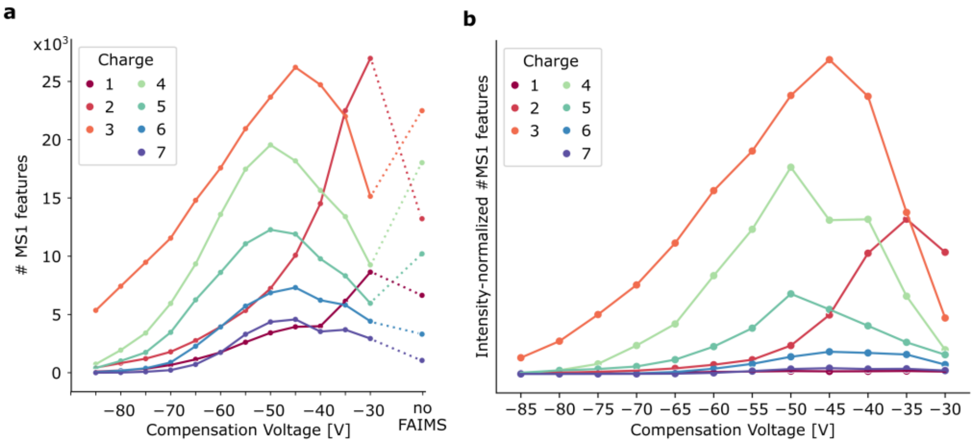


**Figure S-6: Distribution of MS1-features in dependence on CV during FAIMS separation with 26S*BS3 sample**

Distribution of MS1 features by charge in dependence on selected FAIMS compensation voltage with the BS3-crosslinked 26S proteasome sample. The plot in (a) gives total numbers while in plot (b) MS1 features were normalized by intensity. Note that there is a marked decline in intensity when applying more negative compensation voltages.

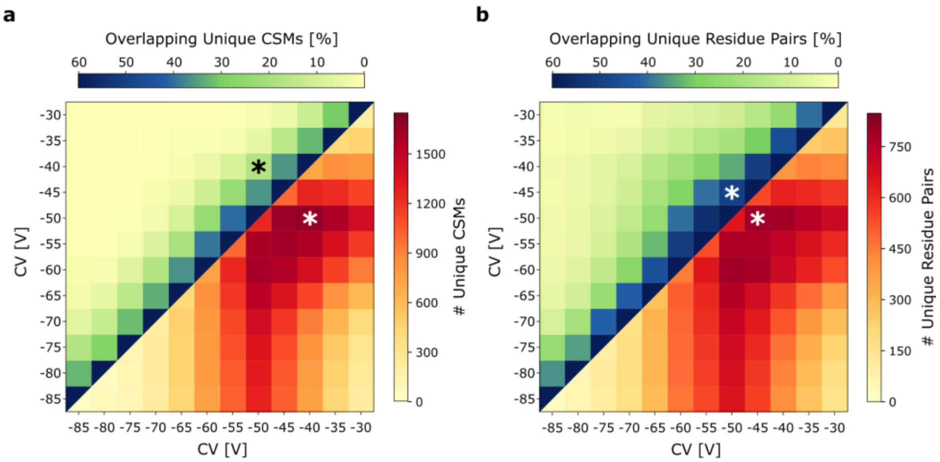


**Figure S-7: Optimal CV pair for unique CSM and residue pair detection for all SEC fractions of 26S*BS3 sample**

The heat maps show the results from combining two compensation voltages in-silico on the detected number of unique CSMs (a) or residue pairs (b) and their corresponding overlaps for the 26S proteasome crosslinking experiment, globally for all studied SEC fractions. Asterisks (white or black) mark the best combination of two CV values on both sections of the plot.

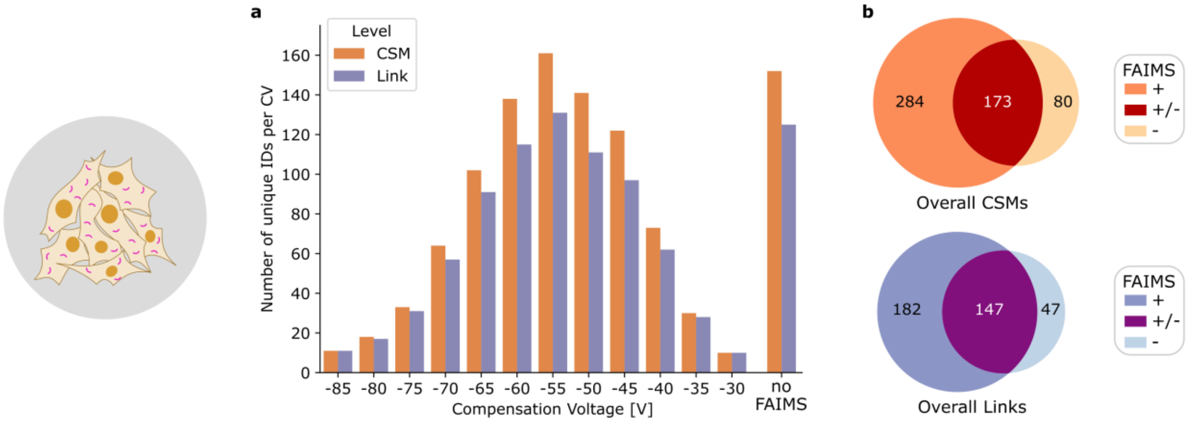


**Figure S-8: Unique CSMs and links from +/-FAIMS for 293T*DSSO sample in dependence of CV and in total**

(a) FAIMS separation of DSSO-crosslinked peptides from whole-cell crosslinked 293T cells displays a unimodal relationship between detectable CSMs and compensation voltage with an optimum at -55 V. Each bar represents the outcome from a single LC-FAIMS-MS analysis considering one CV or the median from triplicate LC-MS analyses of two hour analysis time both, respectively. Using FAIMS led to slightly more detected CSMs and crosslinked residue pairs than not using it. (b) Venn diagrams on overall uniquely detected CSMs and links with or without using FAIMS. Abbreviation: CSMs = crosslinked spectrum matches.

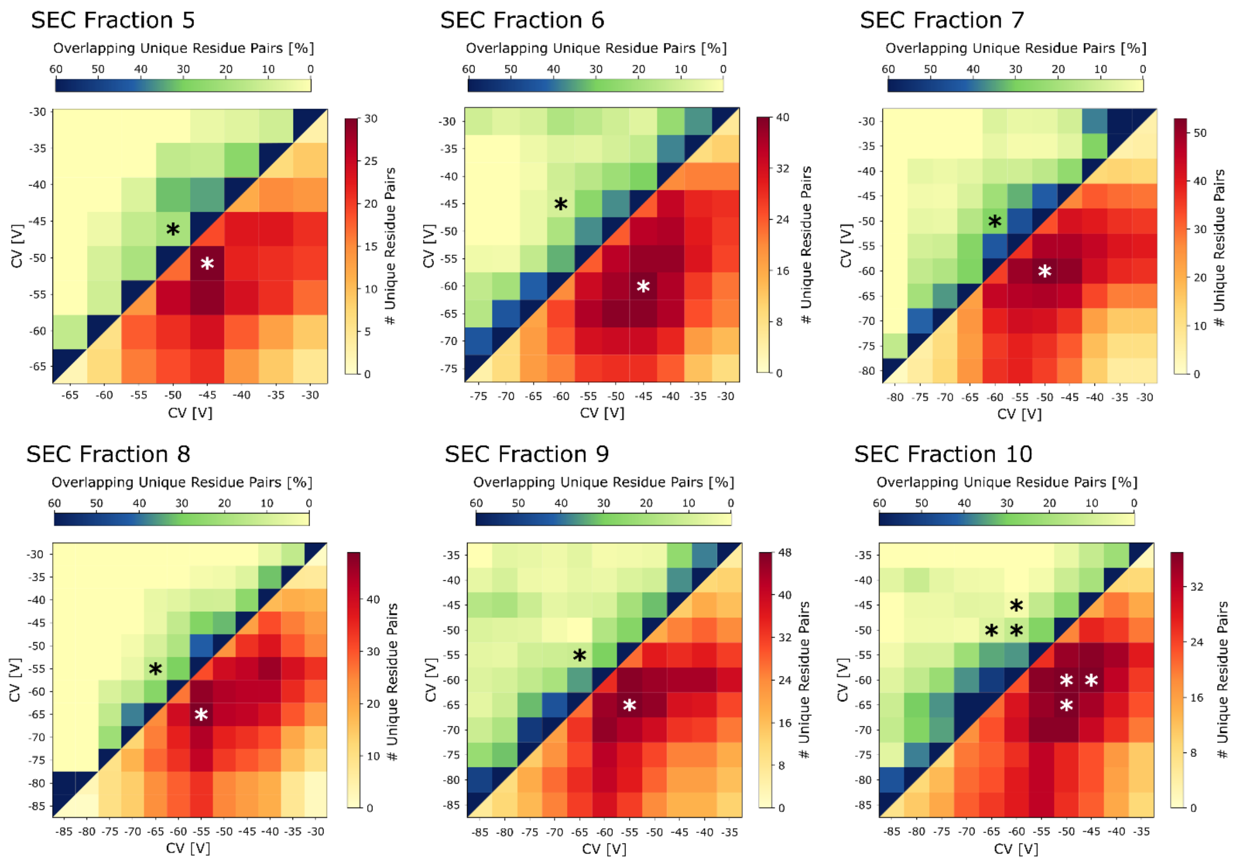


**Figure S-9: Optimal CV pairs for individual SEC fractions of 293T*DSSO sample**

The heat maps show the result from combining two compensation voltages in-silico on the detected number of unique residue pairs and their corresponding overlaps for the in-vivo crosslinking experiment, split by each SEC fraction. Asterisks (white or black) mark the best combination(s) of two CV values on both sections of the plot.

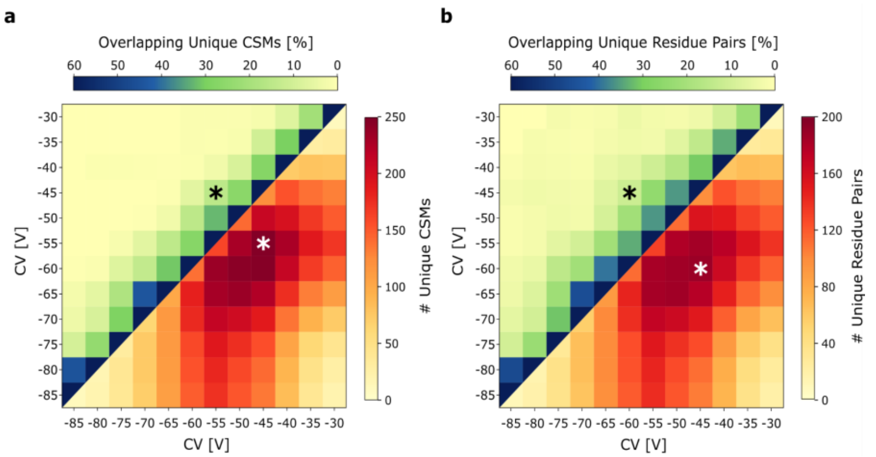


**Figure S-10: Optimal CV pair for unique CSM and residue pair detection for all SEC fractions of 293T*DSSO sample**

The heat maps show the results from combining two compensation voltages in-silico on the detected number of unique CSMs (a) or residue pairs (b) and their corresponding overlaps for the in-vivo crosslinking experiment, globally for all studied SEC fractions. Asterisks (white or black) mark the best combination of two CV values on both sections of the plot.

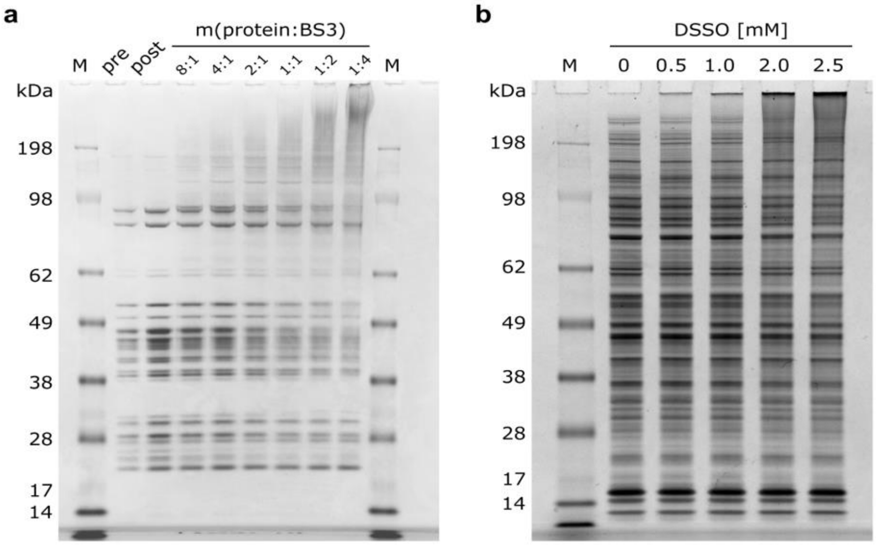


**Figure S-11: Crosslinker titrations with 26S*BS3 and 293T*DSSO**
Results from crosslinker titration pre-experiment to determine suitable crosslinking conditions. (a) Crosslinker titration visualised by SDS-PAGE shows 26S proteasome preparations before ("pre") and after buffer-exchange via spin-filtration ("post"), as well as titration with increasing amounts of BS3 crosslinker. The chosen weight ratio for subsequent FAIMS experiments was 1:3.2. (b) Crosslinker titration for whole-cell crosslinking of HEK293T cells with increasing amounts of DSSO crosslinker, visualised by SDS-PAGE. The chosen concentration for subsequent FAIMS experiments was 2 mM. Molecular weights of the protein standard are indicated at the left side of each gel image.

**Table S-1: XGBoost regression parameters used in a 3-fold cross-validation grid search**

| **variable** | **parameters** |
|---|---|
| n_estimators | 50, 100 |
| max_depth | 3, 7, 9 |
| min_child_weight | 0.1, 1, 10 |
| Learning_rate | 0.1, 0.3 |
| Gamma | 0, 0.1 |
| Reg_alpha | 0.01, 0.1 |
| Reg_lambda | 0.01, 0.1 |
| Subsample | 0.5, 0.8 |
| Colsample_bytree | 0.5, 0.8 |
| Seed | 42 |

**Table S-2: Feature set for supervised machine learning**

| feature name | description |
|---|---|
| length1 | length alpha peptide |
| length2 | length beta peptide |
| length1+length2 | sum of lengths alpha & beta peptide |
| mass | peptide mass |
| loop | 1/0 if loop modification in one of the two peptides |
| oh | 1/0 if oh modification in one of the two peptides |
| nh2 | 1/0 if nh2 modification in one of the two peptides |
| p.charge | precursor charge, detected from mass spectrometer |
| aromatics | count aromatic residues in sequence (F, Y, W) |
| helix | percentage of amino acids favouring helices (biopython) |
| sheet | percentage of amino acids favouring sheets (biopython) |
| turn | percentage of amino acids favouring turns (biopython) |
| pi | computed isoelectric point (pyteomics) |
| charge_glob | computed charge at pH 2.8 (modlAMP) |
| charge_density | computed charge at pH 2.8 (modlAMP) |
| hydrophobic_ratio | global hydrophobicity descriptor (modlAMP) |
| intrinsic_size_sum* | sum of intrinsic size values for all residues |
| intrinsic_size_std* | standard deviation of intrinsic size values for all residues |
| intrinsic_size_max* | maximum of intrinsic size values for all residues |
| mv_sum* | sum of molecular volume values for all residues |
| mv_size_std* | standard deviation of molecular volume values for all residues |
| mv_size_max* | maximum of molecular volume values for all residues |
| polarity_sum | sum of polarity values for all residues |
| polarity_size_std* | standard deviation of polarity values for all residues |
| polarity_size_max* | maximum of polarity values for all residues |
| secondstruc_sum* | sum of secondary structure values for all residues |
| secondstruc_size_std* | standard deviation of secondary structure values for all residues |

| | |
|---|---|
| secondstruc_size_max* | maximum of secondary structure values for all residues |
| estatic_size_sum* | sum of electrostatic charge values from all residues |
| sstatic_size_std* | standard deviation of electrostatic charge values from all residues |
| static_size_max* | maximum of electrostatic charge values from all residues |

Features used in the CV prediction for crosslinked peptides. Features with * indicate summary statistics from custom amino acid tables describing the respective feature[4,5]. Details are available on github.

## References


(1) Schnirch, L.; Nadler-Holly, M.; Siao, S.-W.; Frese, C. K.; Viner, R.; Liu, F. *Anal. Chem.* **2020**, *92* (15), 10495–10503.

(2) Pfammatter, S.; Bonneil, E.; McManus, F. P.; Thibault, P. *J. Am. Soc. Mass Spectrom.* **2018**, *29* (6), 1111–1124.

(3) Chang, C.-H.; Yeung, D.; Spicer, V.; Ogata, K.; Krokhin, O.; Ishihama, Y. *J. Proteome Res*. **2021**, https://doi.org/10.1021/acs.jproteome.1c00185.

(4) Wang, P.; Hu, L.; Liu, G.; Jiang, N.; Chen, X.; Xu, J.; Zheng, W.; Li, L.; Tan, M.; Chen, Z.; Song, H.; Cai, Y.-D.; Chou, K.-C. Prediction of Antimicrobial Peptides Based on Sequence Alignment and Feature Selection Methods. *PLoS One* **2011**, 6 (4), e18476.

(5) Valentine, S. J.; Ewing, M. A.; Dilger, J. M.; Glover, M. S.; Geromanos, S.; Hughes, C.; Clemmer, D.E. Using Ion Mobility Data to Improve Peptide Identification: Intrinsic Amino Acid Size Parameters. *J. Proteome Res*. **2011**, 10 (5), 2318–2329.
